# Supplementary material for: Comprehension of the Route for the Synthesis of Co/Fe LDHs via the Method of Coprecipitation with Varying pH
Source: Nanomaterials (Basel). 2022 May 6;12(9):1570. doi: 10.3390/nano12091570 (PMC9103787; doi:10.3390/nano12091570)
Supplement: Supplementary file 1 [file nanomaterials-12-01570-s001.zip › nanomaterials-1644492-supplementary.pdf]

Supplementary information

Comprehension of the Route for the Synthesis of Co/Fe LDHs via the Method of Coprecipitation with Varying pH

Chérif Morcos [1,2], Alain Seron [1,*], Nicolas Maubec [1], Ioannis Ignatiadis [1] and Stéphanie Betelu [1,*]

[1] BRGM, French Geological Survey, 3 Avenue Claude Guillemin, CEDEX 02, 45060 Orleans, France;
c.morcos@brgm.fr (C.M.); n.maubec@brgm.fr (N.M.); i.ignatiadis@brgm.fr (I.I.)
[2] LGC, Chemical Engineering Laboratory, University of Toulouse III, 118 Route de Narbonne, CEDEX 09, 31062 Toulouse, France
* Correspondence: a.seron@brgm.fr (A.S.); s.betelu@brgm.fr (S.B.)

Geochemical Modelling

The PHREEQC® geochemical code was used for thermodynamic investigation based on the associated THERMODEM® thermodynamic database generated by BRGM. The geochemical model predicted the evolution of the species concentration during the modeled titration of dissolved $Co(NO_3)_2$ (0.15 M) and $Fe(NO_3)_3$ (0.05 M) by the alkaline titrant solution of NaOH (2.80 M) and Na_2CO_3 (0.78 M) at 35 °C, according to equilibria with ferrihydrite 2 line, ferrihydrite 6 line and $Co(OH)_2$ (Figure S1). The conductivity values were calculated according to the following formula:

Conductivity (at T °C) = ([C] x $\Lambda°m$) x (1 + 0.02 x (T - 25))

where [C] is the concentration of the involved species in $mol.m^{-3}$, $\Lambda°m$ is the value of the ionic conductivity at 25 °C and at infinite dilution ($\Omega^{-1}.m^2.mol^{-1}$) and T is the temperature in °C.

The shape of the modeled conductivity is not in agreement with data measured during the experimental titration. Indeed, the conducted modeling does not take into account the thermodynamic data related to adsorption of Co^{2+} on ferrihydrite and Na^+ on precipitates as well as coprecipitation of LDHs, because these data are absent in the database used. However, the intercomparison between experimental and modeled titrations enabled interpretation of the different phenomena that take place during the experimental titration.

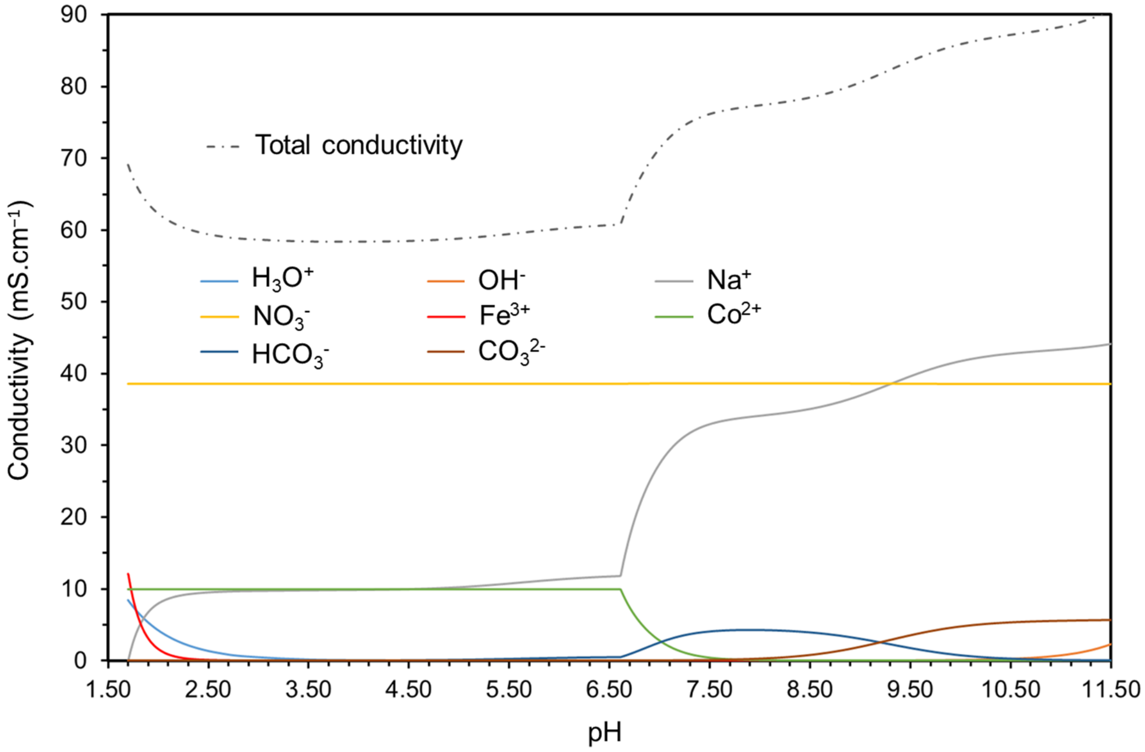

Figure S1. Modelled evolution of conductivity versus pH values during the modelled titration of dissolved $Co(NO_3)_2$ (0.15 M) and $Fe(NO_3)_3$ (0.05 M) by the alkaline titrant solution of NaOH (2.80 M) and Na_2CO_3 (0.78 M) at 35 °C, according to equilibria with ferrihydrite 2 line, ferrihydrite 6 line and $Co(OH)_2$.

pH and Co^{2+} concentration versus time

As the synthesis procedure has consisted in a linear adding of an alkaline aqueous solution versus time into the cationic solution inducing pH increase until reaching the desired value, data provided from Co^{2+} concentrations obtained by SAA and calculated by using conductivity measurements were used to establish the evolution of Co^{2+} concentration in the reactive solution versus time in the pH range 1.7-8.

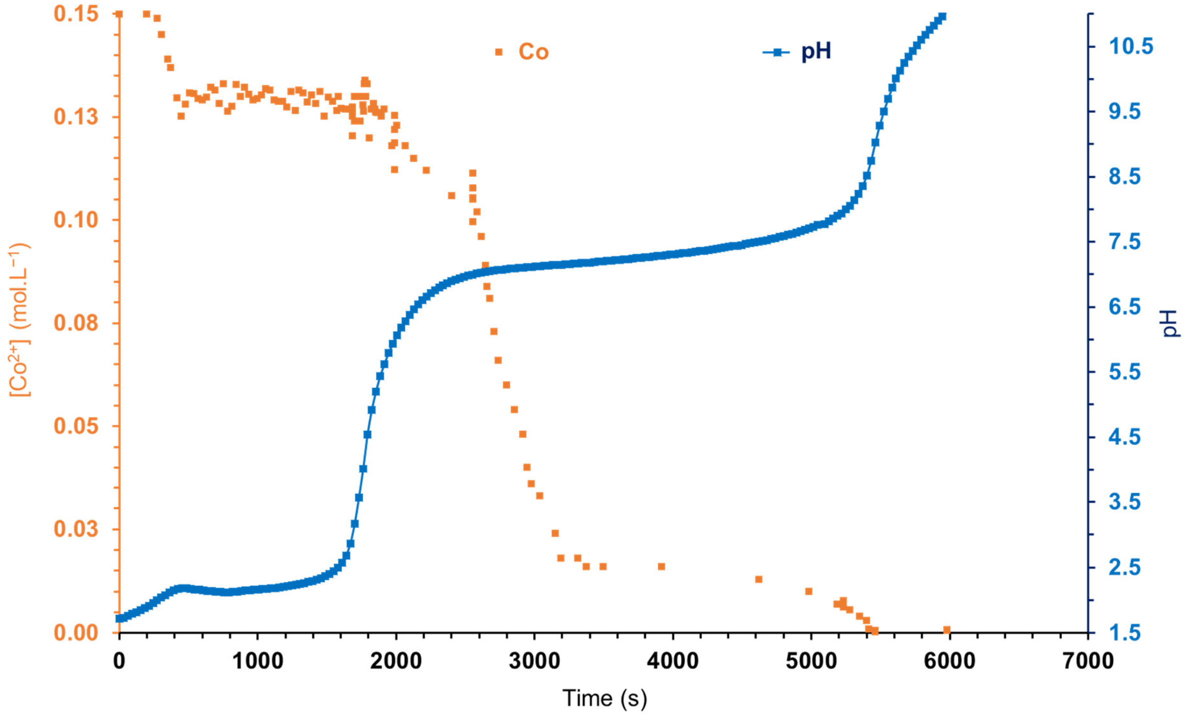

Figure S2. Evolution, versus time, of measured pH and Co^{2+}concentration provided by AAS and calculated using conductivity measurement.

Rietveld refinement analyses

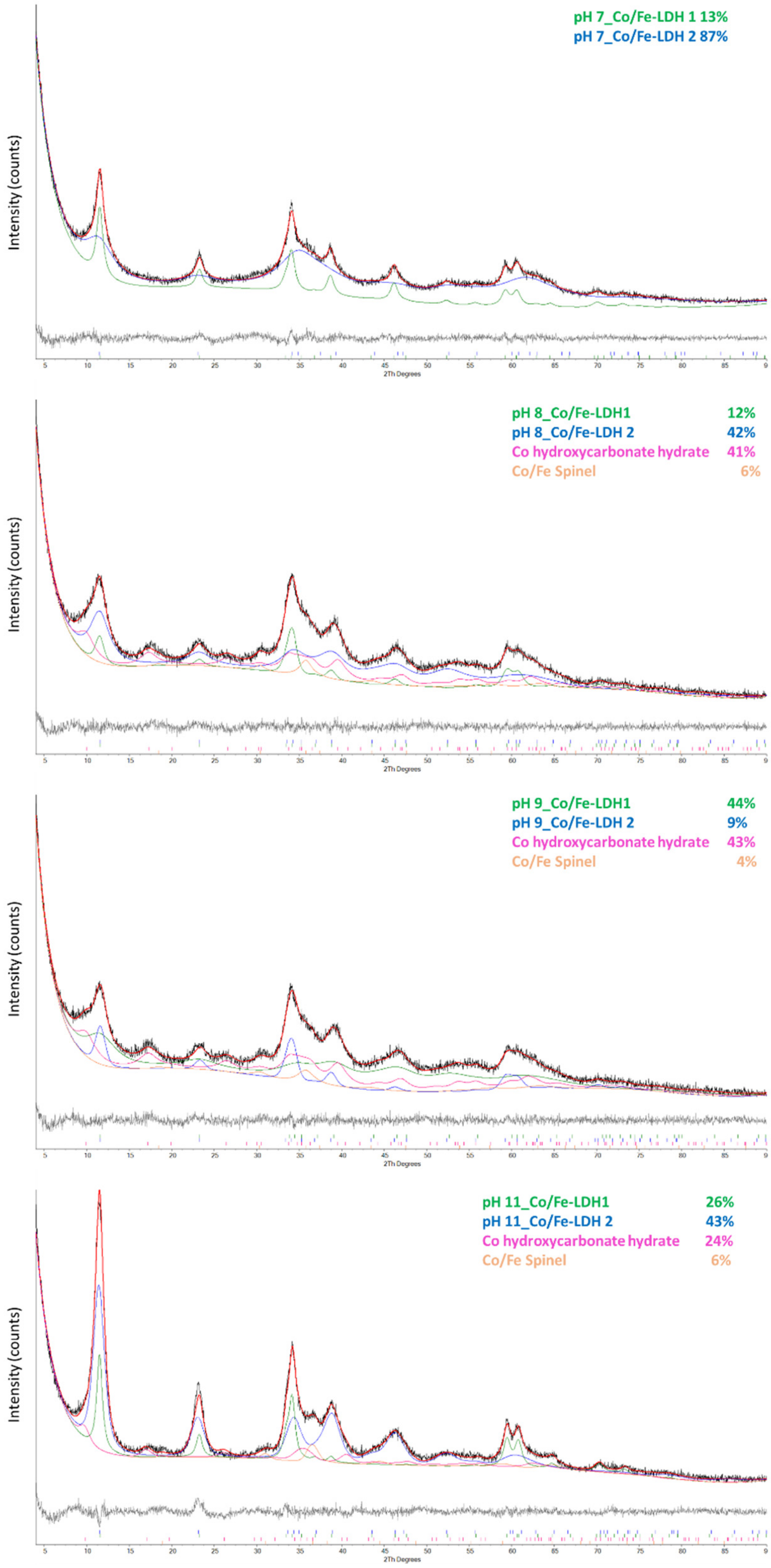

Figure S3. Rietveld refinement analyses of XRD patterns of solids synthetized at pH 7, 8, 9 and 11.

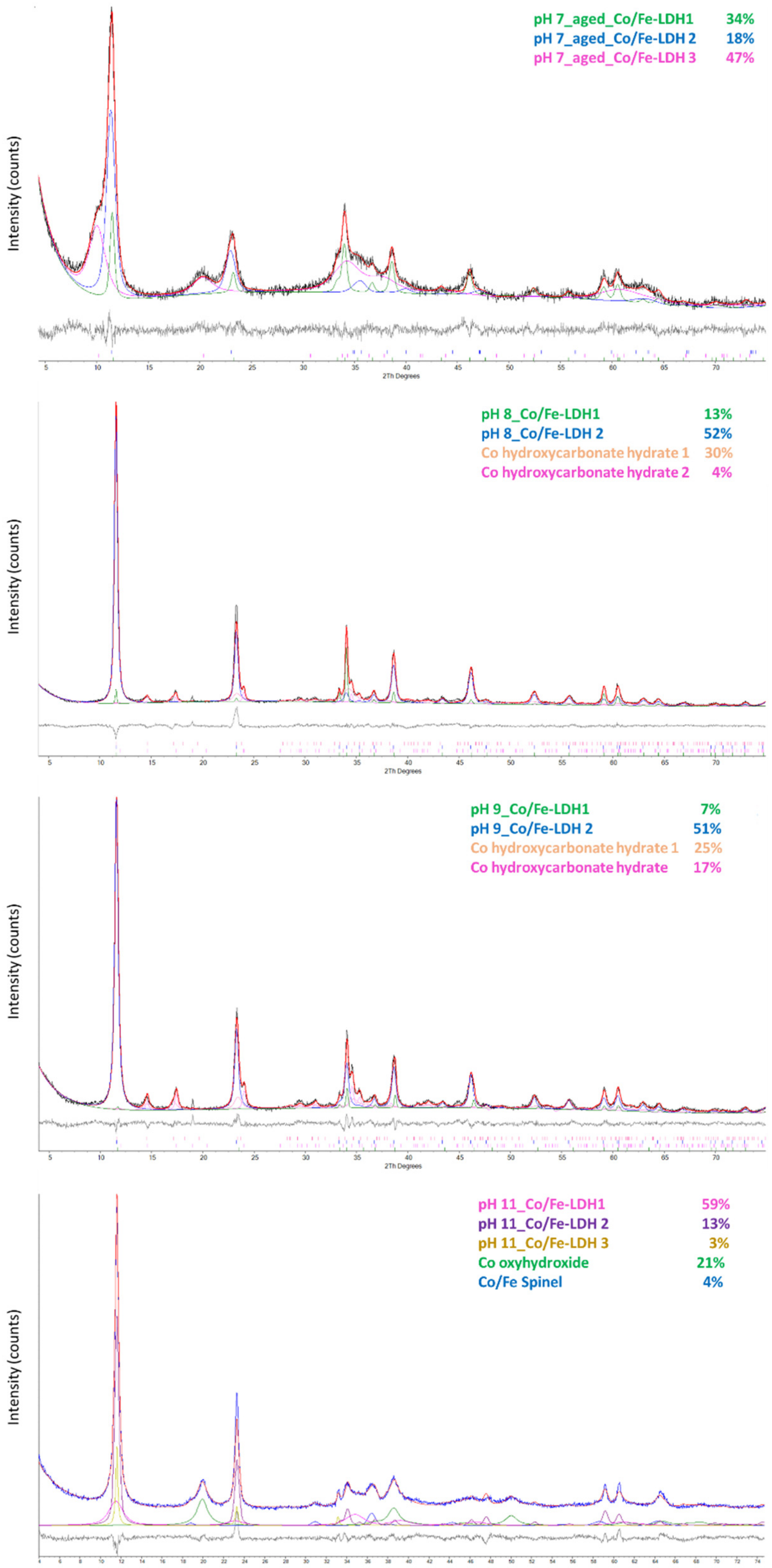

Figure S4. Rietveld refinement analyses of XRD patterns of solids collected after ageing.
